# Protecting Negligence Claimants’ Decisions: An Argument of Doctrinal Coherence in Non-pecuniary Loss

**DOI:** 10.1093/ojls/gqaf025

**Published:** 2025-07-30

**Authors:** Andrew J Bell

**Keywords:** negligence, personal injury, non-pecuniary loss, damages, choice

## Abstract

Various heads of non-pecuniary loss recovery in negligence cast doubt on the explanatory capacity of the traditional twin categories of pain and suffering and loss of amenity. This includes, in particular, loss of congenial employment and loss of reproductive autonomy. The central arguments of this piece are that we can construct from these, based on the existing law, a third category of non-pecuniary loss for personal injury; and that recognising this allows us to rationalise, expand and develop the claims more coherently, rather than castigating them as exceptional extras. The article demonstrates that, alongside pain and suffering and losses of amenity, the courts have already accepted ‘loss of a protected decision’ in these contexts. From that base, the argument considers with more conceptual coherence whether further instances of this category can be accepted in the healthcare and other contexts.

## 1. Introduction

A core tenet of damages practice is a twofold division of non-pecuniary loss in personal injury cases into pain and suffering and loss of amenity (PS and LA, together PSLA). Though any theorised law of personal injury damages only emerged in the 20th century,^1^ this division had established itself by 1964.^2^ Nevertheless, some heads of recovery awarded in practice cast doubt on the explanatory capacity of that framework. This includes the underappreciated loss of congenial employment (LCE), as well as more recent novelties like loss of reproductive autonomy (LRA). The central arguments of this piece are that we can construct from these, based on the existing law, a proposal for a third category of non-pecuniary loss for personal injury; and that adopting this allows us to begin to rationalise and develop the claims more coherently, rather than castigating them as *ad hoc* extras. Alongside pain and suffering, rooted in experienced hedonic impact, and losses of amenity, rooted in non-experiential impacts of bodily injury, I propose that the courts have implicitly accepted what I will term a ‘loss of a protected decision’. From that base, we can also consider with more conceptual coherence whether further instances of this category could be accepted in the healthcare and other contexts, cutting across fraught debates on autonomy-recovery.

This article draws out a proposal for a doctrinal framework for the new category from existing case law on LCE and LRA. It conducts diagnostic work identifying the relevance of choice within those latter areas and related instances of loss, before presenting in measured terms, attentive to countervailing concerns and emerging difficulties, a basis for their further development. The argument frames loss of a protected decision as involving the experience of a factual change which undermines and renders meaningless an established, concrete decision; a previously made choice, held by the courts to be significant to personal status and identity. The article then considers the implications of that structure in medical and other contexts. The broader contention is that arguments for recovery engaging choices in these latter areas could, at the level of recoverable losses, then be viewed, and seen as less controversial, within a much wider umbrella of choice-driven recovery. A doctrinally sound framework for non-pecuniary loss more generally could also be thereby sought.

The endeavour therefore has significant implications for debates over the protection of ‘autonomy’ in negligence and efforts made to secure damages for ‘autonomy’ interference in healthcare contexts.^3^ Often these attempts have confused damage and loss notions, built awkwardly on aspects of the liability analysis (duty and causation), and correspondingly failed to make their case successfully. Several other analyses of the area, meanwhile, have made the radically different suggestion of abandoning ‘loss’ in favour of a rights-vindication approach (or maintain that such an approach has already been taken by the courts).^4^

This article takes a fundamentally different approach. It does not begin with abstract autonomy concepts; it asks more specific doctrinal questions. Nor does it, like a wealth of literature, confront the elements necessary to establish liability; it considers the more under-analysed remedial issue of identifying loss in negligence actions. It asks: how can we understand LCE and LRA; how does their structure account for the central involvement of a claimant’s choice; and in what other situations could that framework be applicable?^5^ From a very specific point of departure, looking at these LCE and LRA losses that have been recognised and compensated for decades, we can take measured further steps towards understanding when there might be compensation related to choice, and in a broader set of circumstances than those as yet contemplated by debates in medical contexts.^6^

In doing this, the article problematises the choice-driven form of these losses, but makes no attempt to justify the life decisions accepted for protection by the courts: the particular decisions chosen for protection will still need explaining under a broader theory. Such a normative discussion is important for enduring acceptance and regularisation of such losses, but cannot be had until the underlying problem is first better uncovered and its parameters within the broader field of non-pecuniary loss better understood. This article thus makes the case for a wider, unrecognised existing extent and form of losses rooted in choice, reorienting existing discussions, but it is not a forum for discussing competing, underlying conceptions of ‘autonomy’ or choice. The argument lies squarely in starting to identify and apply distinctive, unifying features of harms already recognised or recognisable by the courts as losses. That is sufficient to begin a broad new discussion in the field and offer new perspectives on debates in the medical context as the law develops.

One further qualification: the analysis is generally limited to claims based on negligently inflicted personal injury. Many losses that are pecuniary in nature, or claimed outside personal injury in negligence, could conceivably be framed as driven by choice. One example related to some of the discussions here would be the monetary costs of surrogacy arrangements.^7^ Such issues are treated as separable and left aside.

The analysis recaps the divisions between damage and loss and between the two orthodox forms of non-pecuniary losses (section 2), before considering the structure of loss of congenial employment (section 3) and non-pecuniary losses in reproductive contexts (section 4). A short review of the proposed key doctrinal features of those awards follows (section 5). Thereafter, this framework is applied to healthcare (section 6) and wider moral–religious (section 7) decisions.

## 2. Actionable Damage and Recoverable Heads of Losses

I begin with the commonly muddled ‘damage’ and ‘loss’ concepts; the root of serious confusions in existing debates. For the argument below, it is important first to adopt a division between actionable damage and loss.^8^ The two are commonly viewed interchangeably, both involving the claimant being made ‘factually worse off’: a primary harm suffered is designated actionable damage, harms subsequent to it mere ‘consequential’ losses.^9^ However, the concepts are functionally distinct.

Damage is subject to definitional disagreement. Under any definition, however, damage goes to establishing liability. It is a gatekeeper for claims and the locus of a general restriction on recovery for minimal harms—harm of an actionable kind must have occurred.^10^ Negligence liability engages, simply put, when some more than minimal damage is identified as the not-too-remote causal consequence of a breach of a duty of care.^11^ Imagine D carelessly drives at excessive speed, loses control and hits pedestrian C, horribly injuring C’s arm. A claim is made out here (the liability question is answered), insofar as physical personal injury occurs and any non-minimal physical harm to the body is recognised as sufficient damage to ground the action.

This is so before any discussion of the extent of harm suffered and what damages are to be awarded in response (the remedial question). Loss engages with that remedial question—losses for present purposes can be seen as any and all recoverable elements of detriment suffered, with no minimum limit or further restriction within the recognised heads (like restrictions on pure economic losses or psychiatric injury^12^). The function of identifying losses is not to provide gateways to liability, but to describe comprehensively the cognisable harm suffered in order that an appropriate sum in damages can be calculated and awarded as a remedy.

Given this functional separation between establishing liability and determining a remedial response, the definitions of ‘damage’ and ‘loss’ need not match. In fact, damage’s role in gatekeeping liability, rather than describing total harm, suggests an inevitably narrower definition. A simple example of the difference is offered by distress. Whilst distress is recoverable as loss (identifiable with suffering; perhaps termed *consequential* psychiatric harm^13^), it cannot serve as damage sufficient for a negligence claim—for harm sufficient to constitute damage and ground a claim, we require a diagnosable psychiatric condition.^14^

We can disagree about the definition of damage or of any given head of loss, but we should thus be able to say at least that: (i) damage and loss are distinct; (ii) they serve different functions; and (iii) they can be defined differently.

Accepting these features, we should also appreciate that a detriment suffered and identified as ‘damage’ to establish liability may also be *reconsidered* under the different idea of ‘loss’ in order to complete the remedial stage of analysis. In the following paragraphs explicating this, consider the simple example given earlier, where D negligently injures C’s arm in a car accident. Suppose C suffers pain and emotional distress; suffers general inconvenience in their daily life without their dominant arm; and must give up their fulfilling career as a surgeon.

Liability arising will depend on establishing damage of an actionable kind. Whatever one’s preferred definition, this will certainly be satisfied in the example by the first, physical, personal injury: the arm. We do not revisit the ‘damage’ question for pain/distress, inconvenience, and lost employment, etc, applying the gatekeeping standards of damage to the causally consequent elements of detriment: the gatekeeping function is sufficiently served and the claim has been recognised.

Assessing the appropriate sum to award in damages at the remedial stage is a functionally distinct enterprise, requiring us to look at the totality of detriments suffered (filtering down to those recognised by the courts). We must consider all of those involved: the lost arm itself and associated inconvenience (a loss of amenity), pain/distress (pain and suffering), lost pay (loss of earnings), etc. On the basis of the fullest possible breakdown of the detriments experienced, the courts will be able assess sums appropriate to award as damages.

Now, it is clear that the root personal injury (the injured arm) has appeared twice: once to satisfy the damage requirement, and again when describing detriment for remedial purposes. Usually, we would look into such a first-detriment-in-time only as ‘damage’ (establishing liability), without also explicitly framing it as ‘loss’ in a separate remedy-assessing sense—we might talk of assessing an award ‘for the damage’ and other ‘consequential’ losses. Such shorthand expressions are generally unproblematic, but nevertheless analytically confused. Loss must be separated off as suggested, because an accurate and comprehensive depiction of the claimant’s overall harm requires that losses are defined consistently with one another and do not overlap (avoiding overcompensation).^15^ This need for consistency and non-overlap means that definitions of different losses cannot be dependent on the causal circumstance of their occurring chronologically first or later (‘consequentially’)—identifying something as ‘damage’ is not just placing a different label (using more restrictive criteria) on the same thing as ‘loss’ for the first harm that occurred. The situation is quite different from the liability question of what constitutes damage—many gateways may be open, and it does not matter which we enter. Damage’s functionally distinct operation means that it can, however, be defined differently from any given loss.

With that understanding in place, we can turn to loss; the elements of describing the totality of detriment. On the internal features of the loss concept, it has been consistently held that non-pecuniary losses in personal injury cases are of two kinds, even if singular sums are often awarded to include both: PS and LA.^16^ The former consists in the hedonic impacts felt: it is understood ‘only [to] exist by being felt or thought or experienced’ by the claimant.^17^ Where experience cannot have been possible, there can be no recovery under that head. By contrast, a loss of amenity exists in an injury itself and the inconveniences that flow objectively from it, regardless of awareness; even claimants who never know of their condition recover LA.^18^ In the paradigm cases of unconscious claimants, damages can thus be recovered for physical losses of amenity, but not experience-based pain and suffering.^19^

The division can seem intuitive, but it provokes significant disagreement—the dissenting speeches in the seminal *West v Shephard* decision that entrenched it themselves remain powerful counter-arguments.^20^ There are many who explicitly oppose it or support alternative understandings, especially on the basis that all non-pecuniary loss must rest in emotional impact.^21^ Certainly, other jurisdictions reject the English dichotomy because of such criticisms, including Australia.^22^ The debate has often played out by reference specifically to unconscious-claimant cases, where the practical uselessness to the claimant themselves of any damages beyond pecuniary losses related to care, etc, are thought to militate against recognising LA.^23^ It is helpful, though, to consider also a hypothetical emotionless psychopath or ideal stoic, for whom the objective non-pecuniary disadvantages of injury would be real and the award usable even without any notable emotional response to the loss. It is thus assumed here that non-pecuniary loss cannot be collated under a singular head related to feeling; there is meaning in framing separate non-pecuniary heads of recovery, including certainly loss of amenity in England.

Accepting the committed position of the English courts and a division between heads of non-pecuniary loss, there is, however, still a problem. Some losses are well recognised and compensated despite not fitting that twofold scheme, making at least some reconfiguration necessary. LCE and LRA awards present problems in this respect.^24^ Many view all such awards beyond the *West* dichotomy sceptically. McGregor referred exclusively to PSLA as representing the recoverable heads of non-pecuniary loss; other categories were identified only as ‘Other possible heads’, and that author’s view was that awarding separate sums on the basis of such claimed heads was regrettable and unconvincing in principle.^25^ However, the English courts have not moved further in their practice to reconcile these newer awards with older orthodoxy, nor resolved the resultant conceptual difficulty. Some additional framework is required.

## 3. Loss of Congenial Employment

In order to start to identify a framework of that kind, we can look at the best established of the outliers, LCE. An area where there is surprisingly little discussion, this is a head of non-pecuniary loss long recovered without fanfare. It is doubtless overlooked in part because the loss will generally not arise in a tortured, controversial field (contrast LRA); within personal injury negligence claims, the underlying liability generally clearly rests on damage in simple physical injury. Take the example above of a surgeon’s lost arm: the fulfilling-career loss might well attract an LCE award, consequential on the unexceptional physical injury.

The development of separate LCE awards might be as old as the PSLA dichotomy with which it is inconsistent. McGregor^26^ traces the point to *Hale v London Underground*, which asserted that recovery under the head was well established.^27^ A key historical marker may lie in the late 1960s, when the compensability of ‘loss of a craft’ was being expressly emphasised and said often to be overlooked as a head of recovery.^28^ Some cases today emphasise an origin with skilled vocational workers.^29^

No matter LCE’s origins, an award can now be made where a claimant has been prevented from keeping or embarking on a career of special importance to them.^30^ A particular, acute significance of the employment to the claimant themselves is required and must be evidenced.^31^ For example, in *Hale*, the claimant was a firefighter psychiatrically harmed and forced from operational work after attending the 1987 King’s Cross fire. Alongside other sums, he received damages for LCE of £5000. Otton J emphasised the ‘strong sense of devotion to public duty and the desire to help others that motivate firemen’, and the ‘considerable feeling of fulfilment and satisfaction to attend a fire, to extinguish it … and to rescue any persons inside the building’, which was ‘a real loss’ to Hale.^32^

To the extent that the nature of the detriment is discussed, it is not always easy to pin down precisely. It has sometimes been suggested that the loss is pecuniary, but this cannot be true.^33^ LCE is separated from, for example, loss of earnings and working capacity,^34^ and there is simply no further calculable or estimable pecuniary detriment alleged. No clear assessment principles have emerged for the sums awarded either, but awards are made to be reasonable and modest—Kennedy LJ noted in *Willbye v Gibbons* that they had never exceeded £10,000.^35^ Modest damages figures assessed (not calculated) to be fair and reasonable do not fit a pecuniary loss-type assessment.

As a non-pecuniary loss, LCE cannot rest in a simple question of injured feelings either. The Court of Appeal has previously described LCE as remedying ‘a particular disappointment’, which might suggest equivalence to the ordinary pain and suffering head, in line with the Law Commission’s view.^36^ However, this cannot be understood simply in those hedonic terms. The particular ‘disappointment’ involved must surely in fact relate more to the disappointed expectation about the claimant’s career, rather than the award remedying ongoing feelings of disappointment. This is not ‘loss of congeniality of employment’, after all. Were this otherwise, it would make little sense for awards to be separated out from other experienced feelings,^37^ though they certainly are.^38^ Like lost expectation of life after the enactment of section 1 of the Administration of Justice Act 1982, LCE could have been folded into pain and suffering like other hedonic effects of injury if it dealt in felt disappointment; concrete disappointment would not be different from the emotional experience of the injury generally. LCE would also, in particular, require no finding as to the special significance of the employment to the claimant—losing a job following injury will involve negative feelings for anyone, just to greater or lesser extents. That restrictive feature of the loss, the special importance of the role needed to claim at all, is not explained if the matter is simply a manifestation of pain and suffering.

Furthermore, if we were focused on concrete ongoing feelings of disappointment, there would always need to be a specific, careful comparison with the congeniality of any new employment, but the cases do not properly establish such a comparison either. In many, a new occupation goes unspecified,^39^ but awards—even relatively sizeable ones—can be made where the claimant admits that their new occupation is challenging and fulfilling.^40^ It is worth recalling, too, that the employment does not yet have to have been undertaken by the claimant—the career can have been merely intended/planned.^41^ This tends against any sense that the award is for ‘congeniality’ in the sense of feelings.

There are certainly further judgments suggesting the opposite. In *Hale*, a lost feeling might seem to be the thrust of the extracts above,^42^ for example, and Otton J noted that the loss was ‘not mitigated by any enjoyment from his present work’, and that Hale’s age and employment prospects prevented a higher award.^43^ These do suggest that the loss is congeniality as an oddly extracted subset of PS. There was a particular imperative to distinguish the new employment in that case, however, insofar as Hale remained *non-operationally* employed with the fire service. For the award to be maintained, it was thus especially important to consider whether the new role matched the fulfilling calling chosen.^44^ As to the idea that the claimant was young enough to proceed to other fulfilling work and so could not receive higher damages,^45^ this might conflict with other decisions,^46^ or might equally still be compatible with a focus on the extent of the claimant’s personal investment up to that point in choosing the employment, not concrete, experienced unhappiness after the change. The point in any event went to assessment of damages, where more guidance certainly seems to be needed.

What, though, if not feeling, may be at the heart of LCE awards? Elsewhere, they are expressly described in terms directly drawing on choices: ‘The Claimant has suffered a loss of his chosen lifestyle and the impact of the loss of status and personal identity is significant …’^47^ That statement needs unpacking, given the two ‘losses’. The most natural reading, given that ‘status and identity’ seem to reflect back to the ‘chosen lifestyle’ portion, would be: there is a loss of the chosen (to them especially important) lifestyle, in the sense of a forced change in career, which lifestyle choice has serious consequences in terms of status and identity. As a detriment, this is easy to grasp. Careers are a central means by which we come to understand and define ourselves in society. Rightly or wrongly, people often draw inferences about an occupation as a neurosurgeon or high-powered business executive, for example, and contrastingly about lower-skilled or lower-status jobs. Careers become bound up in our sense of personal identity within society and our sense of pride or achievement.^48^ This also makes sense if, as suggested above, the origin of such awards was in skilled vocational crafts—acknowledging the implications of a division between skilled and unskilled trades.

This is perhaps most acute where undertakings are understood as a ‘calling’ or to indicate particular traits. Many LCE cases concern armed and emergency services,^49^ culturally often associated with particular characteristics (bravery, service, etc) or intense personal meaning (like a family tradition).^50^ It seems, though, that any special status, or membership within a team, etc, can suffice for an LCE award: for example, a decorator redeployed to clerical work,^51^ or a coach painter forced into spraying cars.^52^

If this expression in terms of a forced change in chosen career path, where there are status and identity implications, accurately encapsulates the detriment involved, and it is not enough to have another fulfilling career in prospect to deny an award, then again this is not like PSLA. Instead, the claimant choice that was made is crucially relevant: LCE exists only by reference to the claimant’s choice as to the career to undertake and with which to identify themselves and their status; not by the entry of a feeling or injury itself. The choice is challenged by a factual change, though one which cannot in itself be seen as a loss: it would be absurd to argue without more that employment-as-something-other-than-a-soldier were a loss.

LCE thus poses an important but underexplored loss question and suggests that, accepting its separate award, we require another non-pecuniary category (beyond PS and LA) in personal injury. Here, importantly, the view to that question is relatively clear: there are no confusions over notions of ‘damage’, or other liability problems in the case law. The damage is generally an underlying physical injury on which the career change straightforwardly follows; duty, breach, etc, relate to that injury.

Finally, LCE also follows a fairly uncomplicated pattern of non-pecuniary loss damages assessment, insofar as there is agreement on a variable, modest figure assessed at the judge’s discretion. Explicit principles for the specifics of assessment are not very clear—the three-sentence assessment discussion in *Hale* offers no real reasoning, for example^53^—and we might wish to see more said about career stage, level of personal investment in undertaking the career, etc. Nevertheless, the form of loss itself has certainly been recognised, and awards are made relatively quietly and unassumingly.

## 4. Reproductive Choices

If we accept that LCE sits in a third category of non-pecuniary losses that is driven by a status-relevant career choice, where any factual change experienced cannot otherwise be understood as a loss, we can turn to LRA as the other awkward head among current recoveries and consider whether it is analogous; whether it too can be explained in terms of status-relevant choices. In this section, I reinterpret LRA in this way, drawing on other reproduction-related recoveries, too.

### A. Loss of Reproductive Autonomy

I start with *Rees v Darlington Memorial Hospital NHS Trust*, which concerned the claim of a visually impaired woman whose sterilisation procedure had been negligently performed. Consequently, she conceived and gave birth. Aside any pecuniary and non-pecuniary loss damages for the pregnancy and birth themselves, the House of Lords awarded (a set figure of) £15,000.^54^ The nature of the loss underlying that remains unclear.^55^ The award has been discussed at length in relation to actionable damage and how liability engages,^56^ but those are not the present concern—whether or not the interference involved can properly qualify as damage or establish liability, the court must be taken also to have considered that it qualified as loss (as a matter of the remedy) to award damages for it.^57^ As discussed above, this article only concerns the latter issue.

To that end, consider the decision’s key formulations for the harm. Lord Millett held that the £15,000 was for denial of an important aspect of the claimant’s autonomy, ‘viz the right to limit the size of [her] family’.^58^ Lord Bingham thought that the ‘real loss suffered’ was ‘that a parent … has been denied … the opportunity to live her life in the way that she wished and planned’.^59^ For Lord Nicholls, the award was to *recognise* that a legal wrong with a far-reaching effect (on the lives of the parents and family) had occurred.^60^ In dissent, Lord Hope alleged incoherence in the ratio for the award proposed by the majority in relation to compensation and the set figure.^61^ This important difficulty, and the remaining problems highlighted in dissent, go to *assessment* and awarding the damages figure, rather than the critical, prior identification of the loss that attracts those damages.^62^ To that extent, little discontent as to loss is actually raised in the speeches, though it is clear that no proper ratio emerges as to the nature/content of the same. Their Lordships say quite different things (which must be taken to deal with both damage and loss^63^), and their agreement can only be taken to extend to the idea that some recoverable loss does exist.^64^ There is a failure to engage with the specific definition of this loss, or non-pecuniary losses more generally. The claimant’s choice to be childless was also clearly and explicitly made relevant throughout, but exactly how remains unclear.

### B. Pregnancy and Birth

Equally of interest in this reproductive field, however, are awards made for physical phenomena associated with a wrongful birth or pregnancy, as established in *McFarlane v Tayside Health Board*.^65^ These look to some extent like compensation for physical injuries, but the law has dealt very carefully with the idea that these normal physical processes are unlike standard physical injury. The courts are clear that these need not be seen as ‘injuries’ in any ordinary sense and that their recognition as detriments requires reference to other circumstances: they are not inherently harmful ‘injuries’, but physical phenomena harmful (and so analogous to injuries) insofar as they are unwanted.^66^ Statements in the courts to that effect are framed in terms of the requirement of damage, but from the perspective of loss, the issue is the same insofar as the choice of a claimant not to have a child has been critical in seeing (defining) a detriment here for which a damages remedy is provided.

Again, the claimant’s choice is explicitly relevant and the courts clearly can construct harm based on such a choice where simple reference to the physical facts would not alone suggest that there is remediable harm. Unlike *Rees*, though, this is not framed as a lost right or opportunity—instead, an otherwise non-detrimental shift in the claimant’s position (subjection to pregnancy/birth/etc) is understood as a detriment insofar as this has at the same time engaged an important (reproductive) choice made by the claimant. This makes for a less unorthodox-looking loss structure based on choice than the various modes used to express LRA. It in fact looks more like the position with LCE discussed above, suggesting again that the relevance of choice is going somewhat under the radar when the doctrinal framework of loss is being discussed—there is a wider than appreciated set of materials to work from outside the PSLA structure.

### C. A Right to Choose and Doctrinal Incoherence

To be able to rely on the reproduction case law for present purposes, however, we must consider how to frame the recoveries here, particularly given the controversy surrounding *Rees* and the notion that recovery protects a right to choose whether to have children. We must see how to understand *Rees* in a way that avoids problems identifiable in that notion.

Elsewhere, such concerns have put paid to a *Rees*-style recovery, as with the Singapore Court of Appeal in *ACB v Thomson Medical*.^67^ There, the claimant had undergone in vitro fertilisation treatment with her husband and bore a child. However, the claimant’s ovum had been fertilised with sperm from an unknown person, rather than her husband, and the racial background of that stranger was not the same as either parent’s. ‘Loss of autonomy’ was rejected, and a very different ‘loss of genetic affinity’ introduced as actionable harm.

Initially, with respect, it must be said that there is confusion in the judgment over damage and loss. A distinction is only drawn, for example, between ‘damage’ as injury to be proven to make out a claim and ‘damages’ as money awarded for proof of *that* injury;^68^ however, it is relatively clear that the focus throughout is in fact actionable damage.^69^ Again, damage is not the present concern, so the argument to a large extent targets a different issue. The court in *ACB* does discuss *Rees*, though, and declines to allow recovery on a similar, ‘autonomy’-related basis given three objections.^70^ Two are of little present interest: one is the uncertain content of ‘autonomy’. To construct an entire loss notion from nothing by reference to autonomy would require a firm theoretical position on what that is. But, as noted, this is not the present exercise. The second concern is over-inclusion: existing recovery limits could be circumvented, because anything could be framed as ‘damage to autonomy’. For present purposes, this just speaks to a need to define and delimit the subject matter of recovery well, rather than rely on ‘autonomy’ simply said.

The key concern, however, is doctrinal coherence. The Singapore Court of Appeal highlighted a number of such difficulties that will bear on the current loss question. First, damage requires an objective detriment in the sense of being made worse off.^71^ Does that mean that a choice-based loss would be incoherent? The argument advanced here should be consistent with a counterfactual idea of loss and meet such concerns, though it is not really clear that this is actually necessary as a matter of loss or recovery (as opposed, perhaps, to damage)—recall that losses need not be defined identically.^72^

Secondly and more importantly, can a right of autonomy be the subject of analysis? The Singapore Court of Appeal distinguished rights-based, vindicatory conceptions of negligence and the dominant compensatory approach centred on ‘consequential losses’,^73^ and stressed that rights might inform, but not themselves form, the subject of analysis.^74^ This is also consistent with my position—assuming it is correct and applies across loss and damage, a choice-based head of loss need only avoid making a right to choose itself the subject of the claim to avoid the criticism. Recovery for the physical effects of birth, as just discussed, for example, could draw on choice without focusing on a right to choose—a change in the claimant’s physical state that is not objectively harm might qualify as loss insofar as it thereby challenges a concrete choice made by the claimant.

Can the same be true for LRA in *Rees*? Objectively, there cannot be harm in a contrast between the claimant’s having and not having a child (as a matter of policy and/or practicality; babies might, for one example rationalisation, need to be seen as a boon^75^), just as there is no injury without more in the physical manifestations of pregnancy. But can recognising the existence of a choice made (to be childless) allow that divergence to be recognised as loss? That idea does not necessarily seem satisfying; the example policy that requires a baby to be seen as a blessing would be undermined if recognising a choice to be childless redefined exactly that. We can approach this the other way around, though: what if the pre-existing choice does not turn the factual divergence into a loss, but the change marks a loss of the decision made? The prior choice (not to bear a child) is namely made meaningless when coupled with the physical changes (albeit they are not objectively problematic) brought about by the defendant’s damaging negligence. Applied to *Rees*, rendering meaningless the decision not to have a child could be a loss insofar as it is combined with a factual change in having the child (although that change represents no non-pecuniary detriment in itself). That would mean that, contrary to the various judicial expressions used, the harm in *Rees* is not in being denied a right, choice or opportunity, or autonomy generally; it is in experiencing a factual change whereby the established, concrete (reproductive) decision made is left meaningless. (LRA is then an unfortunate label.)

The same reframing can be applied to *McFarlane*-type awards—the bodily changes of pregnancy have not become losses, but they reflect that the decision not to undergo them is made meaningless. In that case, for assessment, the harm is analogised to physical injury. That element of analogy cannot hold with LRA, given the policy-based position on having children, so a set figure serves as a substitute. The same can also be applied to LCE: in a case like *Hale*, it is not suddenly a loss to be a fire-prevention officer rather than an operational firefighter, but that change marks the permanent disruption of the decision made as to career.

So much may be said for now for reproduction, then: viewing *Rees*-type and *McFarlane*-type awards as involving similar losses, where a reproductive choice made is crucial, can help us overcome apparent problems of doctrinal coherence and build an analogy to the simpler LCE award. As to loss at least, *Rees* appears an isolated doctrinal outlier only when unnecessarily framed in blunt autonomy terms.

## 5. Review: Key Structural Elements of Choice-Driven Loss

With these existing areas of choice-driven recovery noted, we can recap and isolate the doctrinal structural elements of the loss concept that I suggest can emerge, seeing the connective threads through each instance. Two key features resurface repeatedly: a pre-existing decision made by the claimant, and a physical change marking the permanent prevention of this taking effect. By those two features, the claimant experiences a disjuncture between a life chosen and the life available.^76^ Later, we can look at these in novel areas.

### A. Pre-existing Decision

First, the areas where recovery has been allowed relate to a previously made choice, a concrete past decision, the implementation of which is prevented.^77^ Mr Hale had committed to operational firefighting; Ms Rees to not having children. *Later*, relevant breaches of duty prevented the implementation of these choices.^78^ (Comparably, *ACB* involved the claimant’s prior decision to have a child specifically with her spouse.^79^)

As discussed above, contrary to the varying approaches in *Rees*, the proposed loss idea thus centres on choices made, not autonomy, freedom or capacity to choose. Requiring a concrete, pre-existing choice serves to prevent a claimant from unjustly inflating harm after the fact to secure increased compensation. This would otherwise be a significant risk with harm tied to the claimant’s inner life: wherever we need to rest recovery on C’s assertions about their internal thoughts, we cannot overlook the potential for (innocent or opportunistic) exaggeration or simulation of an impact on a past decision. As we develop the concept for recovery here, we must remain alert to these risks and cautious (and careful in the evidence we accept to prove a decision made).

Pre-existence of the decision is also limiting, offering an argument against recovery in related reproductive situations, like destruction of genetic material, for example, where no such award has been made. In *Yearworth v North Bristol NHS Trust*,^80^ briefly stated, the defendants stored frozen semen of the claimants, who were undergoing treatment that risked infertility; the storage system failed and damaged the samples. The decision recognised property in the frozen material and a claim in bailment. We do not need to confront that doctrinal framing for present purposes, though; we can focus on the underlying scenario and how it might be viewed if it presented as a personal injury negligence action. Keren-Paz has similarly reconsidered the scenario and characterises the claim as one for the lost chance to become a father.^81^ The apparent absence on such facts of any recovery analogous to my proposed choice-driven approach is explicable, however, under my scheme: whether an established choice to have a child exists is critical. If no concrete choice to have a child was provably made prior to the genetic material’s destruction, the case is unlike those recognised by the courts as giving rise to recoverable loss.^82^ A decision to leave open the option to have a child with your own genetic material is also different. It better approximates the established recoveries above, but the argument for it is weaker at the normative level of what decisions warrant protection: deciding to decide later is not the same as making the primary decision. (The matter also specifically concerns now-unachievable *genetic* fatherhood—that may warrant protection, as *ACB* perhaps suggests, but the argument seems weaker than with a total disruption of a choice to [not] become a father.)

The requirement of a pre-existing choice also engages a normatively valuable feature, beyond the questions of doctrinal consistency and integration of allegedly exceptional existing recoveries. Any future will be highly contingent and uncertain, and our choices and decision-making capacity are constrained frequently in innumerable ways. But past choices within controlled areas of key life significance, like reproduction, recognised incrementally by courts, will necessarily be much more limited and certain. Rather than protect a general, expansive and ongoing capacity (some variant of ‘injury to autonomy’), the focus is manifestations of decision making that are limited in scope (to some idea of status-/identity-critical decisions) and time (concretised in the identifiable past).^83^ Normatively, the process of identifying, describing and delimiting the scope in terms of relevant decisions would also be limited, being attuned to (and fostering the construction of) particular ‘human meanings’.^84^ The requirement thus provides a guard against a flood of demands to recover on the basis of choice, and avoids the expansionary potential of other approaches.

### B. Permanent Prevention of Effect

The second element we have seen is factual change marking a permanent prevention of that decision taking effect.^85^ With LCE, the entire point is the permanent future unavailability to the claimant of the chosen career. If they can resume the career, there is only passing inconvenience; a loss of amenity for a limited period.^86^ A neurosurgeon with a broken wrist will temporarily be unable to perform surgery but will remain a neurosurgeon and receive a loss of amenity award that will reflect that inconvenience, among others (alongside PS). A neurosurgeon with amputated hands can never operate again, will no longer be a neurosurgeon and might thus claim LCE (alongside PS, and LA for other general inconveniences of the injury).

LRA awards are likewise made where a claimant’s choice not to have a child cannot take effect and this is permanent. Leaving aside related scenarios,^87^ the claimant will from then on always have a child, even if it is given for adoption and the connection is only biological. This could provide a means to distinguish cases of infertility or injury that prevents procreation caused by negligence, and also cases involving destroyed reproductive material. As suggested above, such claimants can still have children, but will be forced into a different route to achieving this, like adoption or surrogacy. That might itself warrant a separate non-pecuniary loss award (alongside any recoverable pecuniary costs for such arrangements), but the argument will be weaker insofar as it goes specifically to biological parenthood, not parenthood entirely. (There are, of course, risks in establishing an inequality of status between children depending on biological relatedness to their parents.)

## 6. A Failed Expansion? Healthcare

So, if this is to become a coherent doctrinal structure for a choice-driven form of non-pecuniary loss, with controllable and normatively grounded limits, where else might claimants try to claim under it? While LCE, LRA and certain other reproduction-related losses have a foothold, the structural features seen there can emerge elsewhere. The obvious first possibility is wider healthcare contexts, where debate already exists. Several claimants have already sought damages by reference to ‘autonomy’ where medical decision making has been compromised by a failure properly to inform or secure consent from a patient. Such attempts have seen no success, but the arguments have also been inadequately made in relation to the notion of loss, broader doctrine and the potential for novel recovery. In particular, the potential breadth and uncertainty of the awards sought have caused concern; perhaps inevitably where there has been no discussion of loss significant enough to deal with the conceptual challenges that emerge.

This section outlines how claimant representatives have unsuccessfully attempted thus to exploit the opportunity seemingly created by reproductive loss and by other decisions that have drawn on some notion of patient autonomy—chiefly *Rees*,^88^  *Chester*^89^ and *Montgomery*.^90^ It argues that, considering the proposed framework above, existing case law has not yet comprehensively ruled out choice-driven recovery analogous to awards in the reproduction and employment contexts. The proposal has the potential instead to cut through existing debates and the potential for significant future development.

Consider *Diamond v Royal Devon & Exeter NHS Foundation Trust*, where, for present purposes, a surgeon allegedly failed to provide sufficient information concerning a mesh-based hernia repair, which made having further children inadvisable. The High Court found that, properly informed, the claimant would still have undergone surgery.^91^ The claimant submitted, however, that negligent non-disclosure in itself creates a right to damages. The court considered the *Montgomery* and *Chester* decisions’ discussions about respecting patient autonomy,^92^ but noted their limited, tangential relevance. *Montgomery* concerned breach of the information duty;^93^  *Chester* a ‘modest departure from established principles of causation’.^94^ This is unimpeachable in relation to our loss discussion (those liability issues cannot refer to loss), and the court rejected a self-standing claim for the failure to inform.^95^

The presentation of what was actually sought in *Diamond*, however, had remained unclear. HHJ Freedman variously discussed a ‘right … to claim damages’, ‘a free-standing claim in damages’ and a ‘free-standing remedy’,^96^ implying various possibilities. The critical discussion at paragraph 58 concerns actionable damage. The ideas at play are thus inadequately expressed; no clear understanding emerges as to what loss the claimant believed should be compensated. The claim’s rejection does not, therefore, rule out recovery under my proposal.

Subsequently, the Court of Appeal sought to put an end to autonomy-loss arguments in failure-to-inform cases,^97^ supplying broader argument. But those arguments also still leave room for loss of protected decision. In *Shaw v Kovac*,^98^ the claimant brought a negligence action as the representative of her father’s estate, where he had died after a procedure being trialled by the hospital and, properly informed, would have declined the operation. The defendants disputed damages. PSLA damages were awarded, but an additional £50,000 for denial of the right to choose the treatment was denied.^99^ On appeal, the claimant identified no satisfactory precedent for this loss,^100^ but argued for a substantial compensatory award based on *Chester*, *Montgomery* and patient autonomy. The claimant’s alternative submissions sought a ‘conventional’ award for lost personal autonomy by analogy to *Rees*. The appeal was dismissed.

In Davis LJ’s lead judgment, multifarious framings for the potential award are contemplated.^101^ Counsel seemed to have difficulty formulating the claim.^102^ Counsel expressly disclaimed any separate cause of action or vindicatory award,^103^ however, and made limited, secondary arguments for a ‘nominal’ or ‘conventional’ award.^104^ Whilst novel, the claimant’s argument was explicit in where the novelty should fit: recognition of a new form of non-pecuniary loss to be compensated.

The court’s own approach, separating ‘conventional’ and ‘compensatory’ awards, was itself problematic: any non-pecuniary loss award is ‘conventional’ (‘correct’ only by conventional recognition),^105^ and it is not clear that ‘conventional’ awards are any less ‘compensatory’ than non-pecuniary loss awards generally.^106^ The only relevance of that dichotomy—whether the award is a set figure or variable—is for the *assessment* of damages and says nothing for the prior question of whether there was a cognisable loss. Do, then, the arguments against a ‘compensatory’ or ‘conventional’ award prove fatal to opening up the development of loss of a protected decision in my sense?

### A. A ‘Compensatory Award’

Regarding a ‘compensatory award’, Davis LJ relied on two lines of argument. His Lordship maintained that no authority directly supports awarding such damages.^107^ This is fine, but his Lordship also maintained that the proposition is contrary to general legal principle.^108^ This, and whether other precedents support an award, require attention.

First is the argument that the rights in question (the patient’s personal rights and autonomy, however framed) represent the rationale for the doctor’s information duty, so that an additional award is unnecessary and unjustified: damages are simply for PSLA.^109^ This argument does not really work. Whether or not ‘autonomy’ founds the duty says nothing about what constitutes a recoverable loss. It is insufficient to say that PSLA damages can be awarded after breach of an autonomy-derived duty: those losses are not defined by, and do not directly relate to, the detriment being claimed. We can contrast, for example, Lord Bingham’s insistence in *Rees* that a *McFarlane*-type award alone (for pregnancy’s physical effects) would not do justice to the claimant’s lost opportunity to live as she wished.^110^ Ordinary physical injury cases illuminate another part of the problem with the argument: the duty there is justified by the physical integrity interest, but detriments related to that interest are recoverable and are afforded loss status likewise by reference to it. Whether a loss can consist in depriving a patient of ‘informed choice’ (or similar) cannot be answered by referring to duty.

Regarding this aspect of *Shaw*, and seeking to escape problems thereby raised for his arguments, Keren-Paz argues that where ‘injury to autonomy’ occurs alongside (other) personal injury, policy can limit recovery to the latter—the monetary amount for autonomy injury will be small compared to the PSLA.^111^ Unfortunately, that idea must also be rejected. It blends the separate loss-identification and damages-assessment issues, and entails the denial of a substantial award contingently, based on the lottery of whether other loss also occurs.

Meanwhile, if we do not recognise an additional head of loss in situations of the kind at hand, claimants will recover exactly the same in situations where a surgeon fails to inform a patient of an (appropriate) operation’s risks and where it is negligent to undertake the operation at all, and whether or not they informed the patient of risks. All matters relating to the patient’s choices are thus entirely left out of the detriment suffered in *Shaw*, though the choices made are pivotal to understanding the claimant’s core problem. Davis LJ’s note that the claimant is not left without recovery^112^—they get *some* money—fails properly to identify losses and is circular: the novel loss is rejected because there is already a full remedy; only if/because it is rejected can that be said.^113^

Second is the question of the proposed recovery’s scope, and whether it is problematic that loss could arise where an operation was completely successful—his Lordship thought so.^114^ This is also questionable, because the success or failure of the procedure does not relate to the detriment engaged.^115^ The argument rests on a conviction that recovery should never lie for successful operations, but this would mean that a surgeon could be cavalier with a patient as long as they ensure objective success in surgery itself. A patient will less likely want to claim after a success or may not then be able to establish actionable damage, but neither of these undermines any argument for recognition of a *loss* here. There may be a loss which just cannot be claimed because there is no actionable damage, or that is not claimed because the patient has no interest in seeking it.

Relatedly, is an award possible where the claimant would have had the surgery anyway? Again, his Lordship found that impossible to justify.^116^ To the extent that ‘loss of informed healthcare choice’ would be independent of whether the surgery would have happened in the counterfactual, Davis LJ is correct that an award would be possible in result here and this is problematic. My present argument takes a different approach to what is lost, which resolves this difficulty on different reasoning.^117^ The reasoning applied by Davis LJ to the point does not work, with respect. The argument is that consent following non-information is not null. But the fact that consent sufficient to defend trespass exists in either eventuality does not mean that the choice to consent is of the same kind. Again, the argumentation used against recovery does not properly deal with the doctrinal loss issues and so cannot, as claimed, comprehensively dispose of arguments for recognition of a loss rooted in choice.

Finally, assessment: the court was provided no principle to govern a proposed variable-sum award.^118^ Damages assessment is not the present concern, but there are certainly means available to distinguish the significance of different choices. For example, a loss in respect of undergoing life-threatening, experimental surgery would seem to suggest higher damages than one related to a minor, routine procedure.^119^ Such distinctions may not be tenable where (as in *Shaw*) the target is ‘autonomy’ generally,^120^ but that is not fatal to other choice-driven concepts, like mine above.

### B. A ‘Conventional Award’

Thus, *Shaw* did not discuss loss in sufficient detail or provide any fatal argument against substantial compensation for an independent loss in relation to the healthcare choice at issue, whether as defined by the claimant or in the form being suggested above (based on permanent undermining of a prior decision by analogy to LCE etc).

Davis LJ moved on to the possibility of a ‘conventional’ award by analogy to *Rees*. In that regard, bearing in mind the failed differentiation of ‘conventional’ and ‘compensatory’ awards,^121^ it should first be made clear that the courts have authority to ‘create’ an award in the sense of recognising a pleaded detriment as a recoverable loss and awarding damages for it. Past examples have not always gone well,^122^ but the court remains entitled so to ‘create’. Yet the court in *Shaw* was wary, highlighting *Flint*; the *Rees* minority’s concerns; and *McGregor’s* criticism of *Rees*.^123^ Whilst his Lordship rightly did not rest on these misgivings, raising them is telling of his stance.

On the substance, a key concern was whether analogising to *Rees* requires that a court has first declined on policy grounds to award damages otherwise available (pecuniary losses in maintaining the child).^124^ However, the new award cannot rightly be tied to the prior denial of a claim: the unrecovered pecuniary-loss award would never have spoken to the non-pecuniary LRA experienced in *Rees*; LRA must exist independently and regardless of (recovery for) financial loss. This is clear from *Rees*: ‘a modest sum would not, of course, go far towards the costs of bringing up a child … [but] would, however, adequately compensate for the *very different injury* to the parents’ autonomy’.^125^ The award cannot be a consolation prize. The apparent non-recovery for LRA in infertility cases might be taken to support the view that *Rees* does require the claimant to have first been deprived of a different award. However, that unavailability can be explained better in terms of loss by there being other ways to bring children into one’s family and/or having made no specific decision to have a child, as discussed above.^126^

Additionally, scope and floodgates reasoning reared its head; Davis LJ noted that any award might be available for other torts, medical situations or infringements of personal autonomy.^127^ However, *Rees* could already extend well beyond its immediate negligently-performed-sterilisation context. If that did not prevent Rees’s award, why did it Shaw’s? Here, a willingness to frame *Rees* as a singular anomaly creates untenable distinctions, incompatible with any coherent loss concept. The proposed loss structure above would mean that the *Rees* award is actually a sound parallel to *McFarlane* and LCE. The definitional question of loss does not collapse into the unprincipled mire suggested by the approach in *Shaw*.

At this juncture, we might note the impact in *Shaw* of the patient’s death. The claim was treated as an attempt to secure damages for loss of expectation of life, an abolished head of loss.^128^ Ultimately, Davis LJ ‘formed the very decided impression’ that damages were really being sought for lost years of life.^129^ If the loss is properly raised in terms of a decision made, however, the claimant’s initial, improper focus on lost life is no counter-argument.^130^ Moreover, the risk to life was a key qualifier for the nature of the patient’s situation; though death could not count as a loss, the risk of it typified the choice involved (and so might be discussed).

As to a ‘conventional’ award, furthermore, his Lordship highlighted^131^ Lord Hoffmann’s obiter rejection in *Chester* of a ‘modest solatium’ for patients to vindicate their right to choose, because the risks involved can vary in severity, fixing a figure would be very difficult and tort law would not be a cost-effective vehicle to distribute such sums.^132^ These arguments go to damages assessment, not recognition of a form of loss, but note that the variability in the subject of loss simply argues for a variable sum—as for LCE or as in *McFarlane*. As noted, there is a very specific reason for *Rees* to set an invariable figure. Beyond this, any assessment problem involves the inherent difficulty of remedying any non-pecuniary losses.^133^ Again, then, there is nothing said conclusively to prevent a parallel to the recognised recoveries above. Much the same undoes Lord Hoffmann’s costs argument: in terms of cost efficiency, tort is always somewhat problematic as a mechanism for delivering compensation, and particularly for non-pecuniary loss; the argument bites too deep or not at all.^134^

As to an actual damages figure, the claimant in *Shaw* also sought an astonishing £50,000; manifestly too high and over triple the *Rees* LRA award^135^ or the £15,120 available for bereavement.^136^ It is perhaps this sum that made the courts so nervous, so willing to identify a ‘disguised’ claim for death and raise assessment concerns.

Again, though, the court in *Shaw* did not fully argue the question as to loss. There is confusion between loss-identification and damages-assessment principles, and over the issues relevant to understanding loss. Thus, the arguments against recovery in these cases offer no basis to suggest that experiencing a change that permanently disrupts a recognised prior decision cannot coherently represent a recoverable loss in the healthcare context, analogously to LCE and others, as framed above.

### C. A Loss of Protected Decision

So, developments thus far have not sufficiently considered the doctrinal loss issues in the healthcare context, and there has been no exploration of what can count as loss where a past decision is disrupted in a comparable way in other contexts. The law recognises a number of recoveries not contemplated by the *West v Shepard* dichotomy and could make use of those to try and develop more coherence here, in the way that I suggest above: developing a loss-of-a-protected-decision analysis. Some confusion is also apparent, with unfocused and shifting claimant arguments.^137^ Developing a protected-decision analysis could offer more coherence and order here, cutting through existing tangles over ‘autonomy’.

What might be an answer to the question of recovery, then, if we started to develop from the more limited, focused proposal for a loss of a protected decision discussed above? Could we conceivably identify both a relevant prior decision and factual change marking its permanent disruption? And if so, can such a loss ever conceivably be recovered in light of broader constraints, particularly a need for the loss causally to follow from actionable damage inflicted in breach of duty?

It is clear already that (subject to judicial recognition of such decisions as being of a relevant status-/identity-impacting kind) some cases could fit the required structural pattern. If C has made a decision to receive treatment X, not Y, but D, through negligent recordkeeping or similar, accidentally administers X and this cannot be switched back or corrected, there would seem to be some potential for recognising a loss of protected decision insofar as a pre-existing choice is made and is rendered meaningless. Boundary lines could also be drawn between different kinds of choice: for example, between life-and-death or end-of-life medical decisions on one hand and less critical medical treatment choices on the other.^138^ To stress again, though, such normative questions about what choices count are beyond the scope of the present argument; for now, I suggest only that these sorts of choices could start to be accommodated doctrinally by developing a broader framework for understanding choice-driven losses, analogising to existing awards (commitment to career, or reproduction).

However, everything would turn on the specific facts, and there will be issues in various cases with the permanence of the factual change involved; the pre-existence of the decision at issue; and the causal relationship between elements of the claim.

As to permanence, informed consent cases offer good examples: the particular healthcare decision at issue might be permanently or only temporarily prevented from taking effect. However, this is no trickier than with simple personal injury and LCE. Suppose that C must choose between lifelong drug treatments X and Y, where X carries a risk of triggering migraines (wearing off only once the patient stops taking the drug). C would have avoided this at all costs, but D did not properly inform C of the risk. C chose drug X and suffers migraines. If both drugs remain viable treatment options, C will switch to treatment Y, stopping the migraines; C’s decision-consistent life was put on hold and C can claim, if anything, only PSLA. However, if the choice is permanent—each drug excludes the possibility of ever thereafter taking the other—C cannot make the switch and continues to suffer migraines; her decision has been permanently disrupted and (assuming the courts would recognise such a treatment choice as being of the right kind) she might conceivably frame a claim in terms of that additional loss of a protected decision. As long as a court is able to unpack whether a claimant is permanently prevented from giving effect to their choice, rather than merely temporarily, there is no insurmountable difficulty.

Non-disclosure cases also raise questions in terms of the pre-existence of the relevant decision: a treatment decision actually made is not disrupted if C receives the treatment consented to; no other more-informed, non-implemented choice has previously been made. Comparing *Shaw* and *Diamond* to LRA and LCE suggests exactly this boundary between cases of disrupted choice and cases where there is no pre-existing choice to be disrupted.

This is not the end of the matter, though. Where, on the evidence, we can determine what treatment the claimant would have opted for (if properly informed), could that be enough to fulfil the idea of a decision since disrupted? The determination would have to be made with sufficient certainty, based on evidence of other decisions and preferences pre-existing that treatment election. It could also not leave open to the claimant an opportunity after the fact to assert a newly advantageous supposed preference. Subject to these points, medical non-disclosure case choices could then again have the potential to produce recovery under the banner of loss of a protected decision in the sense proposed above. Consider this: C has an unshakeable lifelong conviction that they will not risk death on an operating table, preferring to die at home. D proposes surgery X, carrying a high risk of death, which D should disclose but negligently does not. C agrees to X and dies in theatre. Insofar as we are able to construct the counterfactual scenario where C had knowledge of the risk and did not choose the surgery (based on sufficient evidence of that pre-existing position), we are able to identify C’s choices behind and prior to the actual treatment consent. We could then draw on precisely this for loss purposes: C had made a standing decision not to risk death on an operating table, and only consented to treatment X, inconsistently with that standing decision, having been given the incorrect information available.^139^ If we determine the hypothetical choice in relation to evidence of the claimant’s decision-making process at a relevant past moment, there is no novel danger of allowing a claimant to redefine their harm. There would be no difficulty in recognising a relevant decision as made, which then, combined with the factual change of the operation and death, would give rise to a perfected loss of a protected decision.

The only thing certainly standing in the way of recovery in at least some informed consent cases within the broader, conceptually more coherent framework proposed above is thus a willingness of courts to recognise the particular medical decisions involved as justifying, like career and reproductive choices, this form of protection (seemingly by reference to some status/identity notion). Despite judicial arguments to the contrary discussed above, the way seems to be navigable both structurally and doctrinally.

I have thus far discussed the possibility of loss being constituted by reference to a claimant’s healthcare decision using two core loss features above, and concluded that this is at least conceivable in some cases. In the context of healthcare decisions, three further questions emerge especially clearly: (i) When will such a loss arise? (ii) What actionable damage could establish liability in such cases? (iii) Could there ever be the requisite causal relationship between actionable damage and such a loss? In order to be recoverable in a negligence action, a loss must be causally connected to actionable damage inflicted in breach of a duty of care. In simple LCE cases, damage consists in a physical injury and causal connection to that damage is clear: the injury will have produced the incapacity that makes maintaining the career impossible. But the same simplicity is missing in medical non-disclosure cases, where there is a failure to inform a patient of an operation’s risks, these risks materialise and the patient would otherwise have declined the operation. Here, the relationship between the failure to inform, the choice-driven loss and the liability-grounding actionable damage seems to lose that clean linear structure. The operation is necessarily selected (and apparently the claimant’s decision disrupted) before any liability seems to arise.

We do not have to accept that. Given that an interference with living consistently with the decision made is not cognisable as loss unless permanent, the interference must emerge later than the initial failure to inform the patient. The fact that the claimant can still change their treatment selection, and a doctor could still provide the missing information prompting just that, precludes the loss of protected decision in my sense arising. It is only on reaching a ‘point of no return’ that the treatment selection can be said to be final and the protected decision permanently disrupted. Accordingly, this choice-based form of loss would arise not when a patient elects without proper information to have a particular treatment, but only once that treatment is begun. As long as some interference that qualifies as damage inflicted in breach of duty takes place at or before that commencement point, and insofar as it is this interference which finally makes permanent the disjuncture between the life available and the chosen life, a choice-based loss can still present as a recoverable causal result. But for that interference, the loss would not have arisen, as there would not otherwise have been a permanent and complete interference with living in accordance with the decision.

Such situations may not be common but, regardless, even in the context of medical consent cases, a cognisable loss of protected decision is not a mere theoretical possibility—it could fall within a conceivable claim. Even if limited in number, meanwhile, just as with LRA and *McFarlane* above, clearing a coherently plotted doctrinal path for losses here can still always promote ordered discussion, and help the gatekeeping damage criterion come into better focus if liability appears too restrictive.

Again, though, the particular structure proposed for choice-based recovery offers reasonable limits for its own application and provides doctrinal and argumentative structure and organisation. Forcing a claimant into inconsistency with a standing decision (to be a soldier, remain childless, die at home) is not enough; it must always manifest permanently through a factual change brought about by the defendant, albeit that change does not otherwise itself constitute loss.

Similarly, no recoverable loss will emerge where the object of the original decision is still met. Where the result of the relevant factual change is no different from the ultimate result in the counterfactual, where the claimant’s protected decision is not prevented from taking effect, we cannot say that the claimant is disrupted in relation to that decision. Where a patient not properly informed about a procedure’s risks would otherwise still have had the procedure, there could be no loss of protected decision award. This is another reasonable limit on the potential expansion of recovery. Where Davis LJ insisted in *Shaw* that an award would be unjustifiable if the claimant would have had the surgery anyway, this was correct, but the reasoning—that misinformed consent was valid—was unhelpful.^140^ (In)consistency of the result with the choice made fits better. (Respectfully, his Lordship’s related insistence that a successful operation cannot lead to liability remains misconceived.^141^)

Moving on to remoteness and the scope of the duty, these too could restrict the recovery of a choice-based loss of the kind countenanced above, engaging the suggested structure and allaying floodgate fears. Suppose that the courts recognise end-of-life decisions as choices suitable for protection and that a terminally ill patient resolves to travel to a euthanasia clinic. Scenario one: a negligent third party’s crash injures the patient on the way to the airport; this delays departure until they are too ill to travel and then cannot end their life as desired. Scenario two: a doctor responsible for the patient’s treatment in preparation for departure negligently causes complications; these prevent departure until the patient’s condition has anyway worsened and travel becomes impossible. Scope of the duty and remoteness reasoning would be capable of maintaining that the driver in scenario one would not be responsible for the loss of the protected decision, while the doctor in scenario two conceivably could be. A connection with the particular duty owed could thus be maintained whilst expanding the cognisable forms of lost protected decisions, again making clear that such progress would not open the door to uncontrollable expansion.

The narrow result suggested in that last example can be contrasted with LCE cases, where the duty need not relate specifically to the kind of decision at issue: loss of chosen career is a risk attendant on any physical injury, unlike loss of a chosen end of life. However, LCE comes with another inbuilt restriction, insofar as the claimant must establish the particular importance of the employment choice, whereas end of life would presumably necessarily matter to everyone.

## 7. Further Expansion? Religion and Morality

If the category of loss of a protected decision can at least conceivably, whilst maintaining doctrinal coherence, expand to other healthcare decisions, we can also consider further areas. One area where commentators focused on damage and autonomy have contemplated recovery is religious and moral choice. Relevant scenarios touch on decisions that certainly in some, perhaps cognisable, sense affect status and identity.

Consider negligently causing another to consume food in contravention of their religious code. Such a scenario appeared in *Bhamra v Dubb*, which Keren-Paz, for example, has suggested may be understood as a case where there was an injury to religious autonomy.^142^ Again, Keren-Paz considers this in relation to damage, not loss, so the present analysis must run differently. Nevertheless: in *Bhamra*, the defendant served food containing egg at a Sikh wedding, contrary to religious practice. The deceased died because of their egg allergy. The case principally turned on the defendant’s duty to warn of food allergens and a related breach of duty; the only damage and loss pleaded was conventional personal injury. However, it is easy to see how, at first blush, in such a case one could start to try and build an analogy to the loss of protected decision structure: a choice to refrain from eating egg as an observant Sikh was made and thereafter disrupted, insofar as negligence caused the consumption of egg.

Assuming liability arises,^143^ it is clear that consuming egg is not in itself a detriment, leaving aside the physical allergic reaction; without more, consuming egg is not harm. An experience-driven loss could perhaps occur: distress from realising that a forbidden food was eaten. However, it is easy to see how a choice-driven sense of loss could be contemplated in such cases, insofar as the consumption of egg is a problem for the claimant once, and because, a decision has been made to follow the religious practice and not eat egg. Such a choice seems to impact personal identity and status,^144^ and is challenged through a factual change which does not in itself constitute a loss.

The limits for such a loss are important, though. One pivotal question is the nature of the choice involved—*Bhamra* entails adherence to a clear and specific religious practice, but the analysis might touch other more-or-less-precisely defined religious practices, and numerous further theological or moral positions. As noted, my analysis does not seek a normative outline of decisions which should be recognised and protected by courts, so this will not itself be discussed further.

However, consider again the doctrinal limits that have been discussed above.^145^ These suggest that *Bhamra* could not have entailed recovery, given the *impermanence* of the problem situation: causing someone to eat egg does not involve a permanent disruption to living consistently with the religious choice to refrain. A Sikh who ingests egg can remain an observant Sikh who does not consume egg, though *Bhamra*’s tragic facts obscure the point in that case. If we isolate the religious choice from the physical harm: after consuming egg it remained possible, in whatever portion of the future remained, to again live consistently with that decision.

This will be true of religious choice generally. The readiest potential candidate for recovery is possibly receipt of blood products as a Jehovah’s Witness. Only situations where adherence to a faith becomes permanently impossible through the wrong would be relevant, though, and examples will be rare, if any at all hold good. For example, if D negligently causes C to undergo a change that means C is cast out of their faith—a sect with a one-strike policy for even involuntary breaches of its norms—then a detriment analogous to that in LCE or LRA cases might arise. Short of such a serious scenario, the analogy to LCE and LRA would not hold. This is also where a divergence from Keren-Paz’s argument for recovery in *Bhamra* bites; that argument maintains that ‘the guests were deprived of a meaningful choice whether to consume eggs’,^146^ but focusing on choice, not a choice made, does not analogise to existing, recognised forms of loss and suggests a far wider, less controlled concept.

Again, then: contemplating choice-driven loss is not, doctrinally well framed, opening the floodgates on a swollen river; the proposed framework remains modest and controllable.

## 8. Conclusion

The law in relation to categorising and defining non-pecuniary losses is not satisfactory, and developments in practice have undermined the orthodox PSLA dichotomy. An account is needed of other recognised recoveries involving claimant decision making in order to correct this problem and help the law develop coherently in future. In this vein, Keren-Paz has, for example, argued that the horse has bolted in terms of recovery based on ‘autonomy’: the courts have already provided protection for certain injuries to autonomy and so greater coherence is now required.^147^ The basic sentiment is good, but rather than focusing on ‘injuries to autonomy’ as establishing a negligence liability, my argument has been the very distinct and more measured claim that certain decisions are recognised as generating loss in circumstances which, but for the choice, could not be seen as such. Recovery has long since arrived; despite its absence from the literature, LCE shows this. This should be uncovered and a more coherent framework to describe such non-pecuniary losses sought.

The primary enterprise here has been to demonstrate a viable potential structure for such a form of loss characterised by choices made, based on where this has emerged in negligence cases. This choice-based loss entails, on my proposal, a particular, pre-existing decision made by the claimant, with that choice seemingly needing to fall within limited categories of status- and identity-relevant decision making acknowledged by the courts as deserving protection. The claimant must then be prevented from giving effect to the decision through a correspondingly undesired factual change caused by the defendant. This factual change need not itself represent a recoverable loss, but the disruption must present as a complete and permanent prevention of the decision taking effect. This framework rests on a doctrinally sound, more coherent arrangement of losses currently already recognised.

Other, related analyses, focused on theories of patient autonomy and on the actionable damage issue, have not appreciated the breadth of the problem field or the vital significance of non-pecuniary loss generally. The key conclusion is that, for all the supposedly doctrine-driven denials of the courts, it is possible to put together a relevant loss concept based on current practice. The concept is no merely theoretical proposition, but a form of detriment seen already in several instances and clearly arguable by analogy in others.

The categories of decision currently acknowledged by the courts relate to careers and reproduction, but the structure could be consistent with application, for example, to at least some further healthcare, and perhaps even religious, decisions. The suggestion emerging from the case law is that recognition of individual types of choice depends on their being somehow critical for personal status and identity; self-direction might also be important.^148^ However, the present aim has not been to confront that normative selection—that argument should be had, but it cannot even begin until a fuller range of existing (or doctrinally conceivable) choice-driven awards is considered. It is not enough to construct a neat theory of autonomy for medical decision making. Questions must also be asked, for one immediate, overlooked example, about the law’s generous treatment of lost career choices over primary leisure activities.^149^

Practically, then, we have not yet reached the limits of the loss concept. In relation to claimant decisions, the case law is not devoid of authority on recovery, and many arguments raised in opposition to recovery are questionable. Ultimately, only serious attempts to systematise non-pecuniary losses will allow us to see this debate’s true parameters, and this article has proposed a first step.

## Footnotes

1 D Ibbetson, ‘Tortious Damages at Common Law’ in R Gamauf (ed), *Ausgleich oder Buße als Grundproblem des Schadenersatzrechts von der lex Aquilia bis zur Gegenwart* (Manz Verlag 2017) 107; A Burrows, ‘Damages and Rights’ in D Nolan and A Robertson (eds), *Rights and Private Law* (Hart Publishing 2012) 276.

2
*West v Shephard* [1964] AC 326.

3 See s 6 below.

4 eg A Mulligan, ‘A Vindicatory Approach to Tortious Liability for Mistakes in Assisted Human Reproduction’ (2020) 40 LS 55, 60ff with further references.

5 In this regard, I align with authors such as D Nolan, ‘New Forms of Damage in Negligence’ (2007) 70 MLR 59 in maintaining that actionable damage is indispensable to the negligence action. I also presume that loss is indispensable and seek to develop an element of that concept, leaving aside rights-vindication accounts and similar accounts.

6 cf though T Keren-Paz, ‘Compensating Injury to Autonomy in English Negligence Law: Inconsistent Recognition’ (2018) 26 Med L Rev 585, also moving beyond the medical setting; that work considers autonomy injury as a basis for liability, however, and so cannot claim the same grounding in current recoveries or limited and controllable scope as my proposal, as discussed below.

7
*Whittington Hospital NHS Trust v XX* [2020] UKSC 14.

8 Confusion here is confronted in some analyses; eg S Fulham-McQuillan, ‘Infringement of Autonomy as Damage in Medical Negligence’ (2023) 139 LQR 126, 136.

9 cf eg D Nolan, ‘Rights, Damage and Loss’ (2017) 37 OJLS 255, 257ff; R Stevens, *Torts and Rights* (OUP 2007) 59, 78.

10
*Rothwell v Chemical & Insulating* [2008] 1 AC 281. I use ‘harm’ in a non-technical sense to mean some negative impact, which can be applied to items of actionable damage and of compensable loss.

11 The complexities of the six-part breakdown in *Manchester Building Society v Grant Thornton UK LLP* [2021] UKSC 20 are irrelevant here.

12 See immediately below.

13 eg H Teff, ‘Liability for Negligently Inflicted Psychiatric Harm: Justifications and Boundaries’ (1998) 57 CLJ 91, 100–1; *KR v Bryn Alyn Community* [2003] QB 1441.

14
*Hinz v Berry* [1970] 2 QB 40. On damage and loss diverging, cf French *dommage* and *préjudice*: D Leczykiewicz, ‘Loss and Its Compensation in the Proposed New French Regime of Extra-contractual Liability’ in J-S Borghetti and S Whittaker (eds), *French Civil Liability in Comparative Perspective* (Hart Publishing 2019) 183–93; from the same volume, O Deshayes, ‘The Importance of Terminology in the Law of Civil Liability’, 449–52.

15 That definitions of different losses must be consistent with one another does not mean they must be identical.

16
*Wise v Kaye* [1962] 1 QB 638; *West v Shephard* (n 2); *Lim Poh Choo v Camden & Islington AHA* [1980] AC 174. cf generally J Edelman and others (eds), *McGregor on Damages* (22nd edn, Sweet & Maxwell 2024) (*McGregor on Damages*, 22nd edn) 41-263ff; A Burrows, *Remedies for Torts, Breach of Contract, and Equitable Wrongs* (4th edn, OUP 2019) 235ff.

17
*West v Shephard* (n 2) 349 (Lord Morris). Clarity on subdivisions varies; pain, suffering, distress and anxiety are sometimes treated as separate elements within the PS head—eg *Manning v King’s College Hospital NHS Trust* [2008] EWHC 3008 (QB) [41]. I leave aside statutory bereavement claims.

18
*Lim Poh Choo* (n 16) 188 (Lord Scarman).

19 A claimant receives less than they would if conscious. See the references in n 16.

20
*West v Shephard* (n 2) 359–64 (Lord Devlin).

21 eg Burrows, *Remedies* (n 16) 36. cf also E Descheemaeker, ‘Solatium and Injury to Feelings: Roman Law, English Law and Modern Tort Scholarship’ in H Scott and E Descheemaeker (eds), *Iniuria and the Common Law* (Hart Publishing 2013) 84ff, who rejects the dichotomy, but would not compensate feelings.

22
*Skelton v Collins* (1966) 115 CLR 94.

23 Consider Windeyer J’s discussion in ibid [10]–[13], referencing Scottish and French thought.

24 See *McGregor on Damages*, 22nd edn (n 16) 41-274ff.

25 See H McGregor (ed), *McGregor on Damages* (19th edn, Sweet & Maxwell 2014) 38-055, 38-260–2; new editors maintain this scepticism: *McGregor on Damages*, 22nd edn (n 16) 40-274.

26 See now *McGregor on Damages*, 22nd edn (n 16) 41-275.

27 [1993] PIQR Q30, Q39.

28
*Morris v Johnson Matthey & Co* (1968) 112 SJ 32 (Edmund Davies LJ).

29
*Zeromska-Smith v United Lincolnshire Hospitals NHS Trust* [2019] EWHC 980 (QB) [111].

30 See generally eg *Willbye v Gibbons* [2003] EWCA Civ 372; *Murphy v Ministry of Defence* [2016] EWHC 3 (QB); C Walton and others (eds), *Charlesworth & Percy on Negligence* (15th edn, Sweet & Maxwell 2022) 6-121; *McGregor on Damages*, 22nd edn (n 16) 41-275–6.

31
*Chase International Express v McCrae* [2003] EWCA Civ 505 [21], [23] (Kennedy LJ).

32
*Hale* (n 27) Q39.

33 See the discussion in Law Commission, *Damages for Personal Injury: Non-Pecuniary Loss* (Law Com No 257, 1998) 3.20; contrast the uncertainty in *Charlesworth & Percy* (n 30) 6-121.

34 eg *Chase* (n 31) [5]–[30] considered care/assistance, past and future lost earnings, travel, surgery, labour-market handicap, home maintenance, etc separately from LCE.

35
*Willbye* (n 30) [11].

36 ibid [11] (Kennedy LJ); cf *Cornbill v Turfsoil* [1993] CLY 1540.

37 cf *McGregor on Damages*, 22nd edn (n 16) 41-275.

38
*Chase* (n 31) [22]. Some observers reject separation from PSLA—Law Com No 257 (n 33) 3.20; contrast, though, P Martin, ‘Loss of Congenial Employment’ [2002] 3 JPIL 284, 285–6.

39 See the example case tables given by Martin (n 38).

40 eg *Byers v Brent LBC* [1998] CLY 1645: £7500, classical musician turned youth worker.

41 As in *Willbye* (n 30).

42 See n 32.

43
*Hale* (n 27) Q39-40.

44 cf *Foreman v Williams* [2017] EWHC 3370 (QB): LCE claim abandoned, same job carried out differently.

45
*Hale* (n 27) Q39-40.

46 See n 40.

47
*Murphy* (n 30) [187] (HHJ Code QC); cf *Bradley v Farid* [1995] CLY 1701: loss of status in spraying cars, not painting coaches (like in *Hale*, the distinction needs emphasis, given the jobs’ similarity to uninitiated observers). cf also *Inglis v Ministry of Defence* [2019] EWHC 1153 (QB): Inglis ‘derived a lot of pleasure from his service and … note in particular his comment that it was an “honour and privilege” to represent the Naval Service’.

48 There is much literature on the interaction between career and identity; some relevant aspects are outlined in B Law and others, ‘New Perspectives on Career and Identity in the Contemporary World’ (2002) 30 British Journal of Guidance and Counselling 431; cf also eg S Raeder and G Grote, ‘Career Changes and Identity Continuities—A Contradiction’ in A Brown and others (eds), *Identities at Work* (Springer 2007).

49 For examples, see military: *Murphy* (n 30); *Inglis* (n 47); *Brown v Ministry of Defence* [2006] EWCA Civ 546; nursing: *Knott v Newham Healthcare NHS Trust* [2002] EWHC 2091; and police: *Rowntree v Commissioner of Police for the Metropolis* [2001] Po LR 404.

50 See also Martin (n 38) 286–7; *Charlesworth & Percy* (n 30) 6-121.

51
*Kennedy v Burke* [1995] CLY 1614.

52
*Bradley v Farid* (n 47).

53
*Hale* (n 27) Q39-40.

54
*Rees v Darlington Memorial Hospital NHS Trust* [2003] UKHL 52.

55 Contrast also Mulligan’s (n 4) view that there is no loss question here; however, the discussion (58) confuses the damage and loss concepts.

56 eg C Purshouse, ‘Liability for Lost Autonomy in Negligence: Undermining the Coherence of Tort Law?’ (2015) 22 TLJ 226.

57 See s 2 above.

58
*Rees* (n 54) [123].

59 ibid [8].

60 ibid [17].

61 ibid [70]ff.

62 See in particular ibid [40]ff (Lord Steyn), doubting the court had authority to create a ‘conventional’ (set-figure) remedy.

63 In the confused, truncated fashion discussed above.

64 Again: I assume damage and loss are essential.

65
*McFarlane v Tayside Health Board* [2000] 2 AC 59.

66 ibid [74] (Lord Slynn), [86] (Lord Hope). Extensive literature considers the controversial point, which cannot be reviewed here; see, though, eg S Todd, ‘Accidental Conception and Accident Compensation’ (2012) 28 PN 196; C Witting, ‘Physical Damage in Negligence’ (2002) 61 CLJ 189; Nolan (n 5); J Conaghan, ‘Tort Law and Feminist Critique’ (2003) 56 CLP 175, 190–4.

67
*ACB v Thomson Medical Pte* [2017] SGCA 20.

68 ibid [44].

69 See eg ibid [44], [114] (‘actionable injury’), [123]ff (‘heads of damage’).

70 ibid [116]ff, [120]ff, [123]ff respectively.

71 ibid [120].

72 See especially s 4 below. Consider also S Steel, ‘Damages without Loss’ (2023) 139 LQR 219, 226–9, for whom the counterfactual element is critical to loss in the ‘usual’ sense, but who countenances the possibility of loss in an ‘unusual’ sense or recovery without ‘loss’. Again, my present core argument of coherence in non-pecuniary loss recovery should meet the need for counterfactual definition in defining loss of a protected decision, but would still apply if labelled as creating another ‘unusual’ loss or recovery without loss.

73
*ACB* (n 67) [121]ff.

74 ibid.

75 eg *Rees* (n 54) [108]–[110] (Lord Millett).

76 An experience of disjuncture perhaps comparable to the nothingness experienced, as a real relation, when a friend is absent at an agreed time/place for J-P Sartre, *Being and Nothingness* (HE Barnes tr, Routledge 2003) 33–5. I thank a reviewer for raising this.

77 Contrast Keren-Paz (n 6), focusing on deprivation of choice.

78 cf also Steel (n 72) 227 regarding not-respected choices and damages for infringed ‘rights of control’. I decline to discuss *Chester v Afshar* [2004] UKHL 41 here, to avoid distracting liability issues of damage and causation, but it might seem problematic that the case does not turn on recovering for a concrete, pre-made decision. There is the potential for inconsistency here, but see further ss 6A, 6C below on consent and pre-existing decisions.

79
*ACB* (n 67) [127]ff.

80
*Yearworth v North Bristol NHS Trust* [2009] EWCA Civ 37.

81 Keren-Paz (n 6) 592.

82 Contrast eg Rees’s concrete decision to be childless; perhaps also ACB’s decision to have children specifically with one’s spouse. On the latter, cf Mulligan (n 4) fn 158.

83 There would be some sociological support for recognising highly consequential ‘fateful moments’, where an individual stands at an identity-critical crossroads—A Giddens, *Modernity and Self-Identity* (Polity Press 1991) 113 (I thank an anonymous reviewer for the reference); Giddens’s examples are broader, including exam-taking, but also include, tellingly: career and marriage choices, and selecting educational studies.

84 cf C Taylor, *The Language Animal* (Harvard UP 2016) 22f, 332f. I thank a reviewer for the reference.

85 cf Keren-Paz (n 6) 602ff: irreversibility.

86 eg *Zeromska-Smith* (n 29): could return after two years; no LCE.

87 Being forced into a termination or experiencing a miscarriage because of a wrongful pregnancy warrants separate discussion, alongside and without undermining the *Rees* award.

88 See n 54.

89 See n 78.

90
*Montgomery v Lanarkshire Health Board* [2015] UKSC 11.

91
*Diamond v Royal Devon & Exeter NHS Foundation Trust* [2017] EWHC 1495 (QB).

92 ibid [54]ff.

93 ibid [55].

94 ibid [57].

95 ibid [55], [56]–[59]. On whether *Montgomery*, *Chester* and related decisions can make an argument as to *damage* as an element of *liability* analysis, cf Fulham-McQuillan (n 8).

96
*Diamond* (n 91) [54], [55], [59].

97 cf *Duce v Worcestershire Acute Hospitals NHS Trust* [2018] EWCA Civ 1307 [88] (Leggatt LJ); *Diamond v Royal Devon & Exeter NHS Foundation Trust* [2019] EWCA Civ 585 [34] (Davies LJ).

98
*Shaw v Kovac* [2017] EWCA Civ 1028.

99 See ibid [35], [41], [43]; cf *Shaw v Kovac* [2015] EWHC 3335 (QB).

100
*Shaw* (n 98) [4], [58] (Davis LJ).

101 ibid [48]–[83]. The *rejected novel cause of action* and the defendants’ *admission of liability* mean that damage (and other *liability* questions) were not at issue: the main arguments relate to the remedial stage and so loss; they can say nothing about damage. The issues for decision were whether the proposed form of loss exists (per Davis LJ: no, end of discussion), *then* whether it existed on the facts, *(only) then* whether that loss was causally connected to the admitted liability. With respect, contrast C Purshouse, ‘Autonomy, Affinity, and the Assessment of Damages’ (2018) 26 Med L Rev 675, 681ff; Fulham-McQuillan (n 8) 135–8.

102 The arguments shifted: *Shaw* (n 98) [32]–[43].

103 ibid [49], [55].

104 ibid [56], [58], [75].

105 See eg *Rees* (n 54) [71] (Lord Hope); *Wright v British Railways Board* [1983] 2 AC 733, 777 (Lord Diplock); *Lim Poh Choo* (n 16) 189 (Lord Scarman).

106 See *Shaw* (n 98) [80] (Davis LJ); cf eg *Rushton v National Coal Board* [1953] 1 QB 495, 502 (Romer LJ)—‘notional or theoretical compensation’; A Tettenborn (ed), *The Law of Damages* (LexisNexis Butterworths 2003) 1.36.

107
*Shaw* (n 98) [58]–[66].

108 ibid [67]–[74].

109 ibid [69], [70].

110
*Rees* (n 54) [8].

111 Keren-Paz (n 6) 600.

112
*Shaw* (n 98) [74].

113 cf Keren-Paz (n 6) 600ff, noting the court’s emphasis on an ‘additional remedy’.

114
*Shaw* (n 98) [71].

115 cf also E Jackson, ‘“Informed Consent” to Medical Treatment and the Impotence of Tort’ in SAM McLean (ed), *First Do No Harm* (Ashgate 2006) 275, 284.

116
*Shaw* (n 98) [71].

117 On successful operations and patients still choosing the procedure in a counterfactual, informed state, see s 6C below.

118
*Shaw* (n 98) [72], [73].

119 See Steel (n 72) 241.

120 Purshouse, ‘Liability for Lost Autonomy’ (n 56) 238ff

121 cf above, text to nn 105–6.

122
*Flint v Lovell*’s ‘loss of expectation of life’ is an example since abolished: [1935] 1 KB 354; s 1(1)(a) of the Administration of Justice Act 1982.

123
*Shaw* (n 98) [77]–[79]; from *Rees* (n 54), see especially [45]–[46] (Lord Steyn). But contrast Lord Hope, who recognised that all non-pecuniary loss damages are conventional—his criticisms concerned the propriety of separating special and general damages, and assessment: ibid [71]ff, [75]ff.

124
*Shaw* (n 98) [79].

125
*Rees* (n 54) [123]–[125] (Lord Millett; emphasis added); cf ibid [73] (Lord Hope). Again, Davis LJ focuses only on getting *some* money.

126 See ss 5A, 5B.

127
*Shaw* (n 98) [81]; cf the overinclusiveness concern in *ACB*: s 4C above.

128 Administration of Justice Act 1982, s 1(1)(a); see the High Court decision: (n 99) [21]–[37].

129
*Shaw* (n 98) [73].

130 The lost-years rule is not sidestepped if a different loss arises prior to death. Contrast perhaps a self-standing claim or autonomy interferences as actionable damage; cf to that extent Keren-Paz (n 6) 601.

131
*Shaw* (n 98) [76].

132
*Chester* (n 78) [33], [34].

133 cf Purshouse, ‘Autonomy, Affinity, and the Assessment of Damages’ (n 101) 683–4.

134 Consider Lord Sumption, ‘Abolishing Personal Injuries Law—A Project’ (2018) 34 PN 113.

135 Albeit some find Rees’s award ‘miserly’—Mulligan (n 4) 65. Simultaneously fighting for a controversial novel loss *and* against conventional value standards was surely too ambitious.

136 Fatal Accidents Act 1976, s 1A.

137
*Shaw* (n 98), particularly [47].

138 cf Steel (n 72) 224–9 and 241. Lord Dyson MR recognised life-saving treatment decisions’ different character: *Tracey v Cambridge University Hospitals NHS Foundation Trust* [2014] EWCA Civ 822 [55] (on *Tracey*, cf Keren-Paz (n 6)).

139 On instances where the treatment consented to is still consistent with the standing choice, see just below.

140
*Shaw* (n 98) [71]; see s 6A above, text to nn 116–17.

141 See s 6A above, text to nn 114–15.

142
*Bhamra v Dubb* [2010] EWCA Civ 13; Keren-Paz (n 6) 596ff.

143 With a serious allergic reaction, like in *Bhamra*, this would still turn on duty, breach and remoteness.

144 Again, this is placeholder phrasing for the normative selection of relevant decisions.

145 See ss 3–5 above.

146 Keren-Paz (n 6) 597.

147 Keren-Paz (n 6).

148 cf Steel (n 72) 227, 241.

149 See *Chase* (n 31) [22] (Kennedy LJ): PSLA incorporates lost hobbies, no leisure-based equivalent to LCE; Martin (n 38) 285–6.

